# Supplementary material for: GeneSetCluster 2.0: a comprehensive toolset for summarizing and integrating gene-sets analysis
Source: BMC Bioinformatics. 2025 Aug 21;26:219. doi: 10.1186/s12859-025-06249-3 (PMC12372222; doi:10.1186/s12859-025-06249-3)
Supplement: Supplementary file 1 — Supplementary Material 1 [file 12859_2025_6249_MOESM1_ESM.docx]

**Supplementary Material**

**Table S1.** Seriation methods from the *seriation* R package.

| Algorithm | Method |
| --- | --- |
| Simulated annealing | **ARSA** |
| Branch-and-bound | **BBURCG** |
| Branch-and-bound | **BBWRCG** |
| TSP solver | **TSP** |
| Optimal leaf ordering | **OLO** |
|  | **OLO_single** |
|  | **OLO_average** |
|  | **OLO_complete** |
| Gruvaeus and Wainer | **GW** |
|  | **GW_single** |
|  | **GW_average** |
|  | **GW_complete** |
| MDS | **MDS** |
|  | **MDS_metric** |
|  | **MDS_nonmetric** |
|  | **MDS_angle** |
| Spectral seriation | **Spectral** |
|  | **Spectral_norm** |
| QAP | **QAP_2SUM** |
|  | **QAP_LS** |
|  | **QAP_BAR** |
|  | **QAP_Inertia** |
| Genetic Algorithm | **GA** |
| DendSer | **DendSer** |
| Hierarchical clustering | **HC** |
|  | **HC_single** |
|  | **HC_verage** |
|  | **HC_complete** |
| Rank-two ellipse seriation | **R2E** |
| Sorting Points Into Neighborhoods | **SPIN_NH** |
|  | **SPIN_STS** |
| Visual Assessment of Tendency | **VAT** |

**Table S2.** Average execution time (in seconds) for running the *CombineGeneSets* ten times at different threads (1, 2, 4, 6, 8, and 10). The workstation used for running this experiment has an Apple M1 Pro processor with a 10-core CPU and 16 GB of RAM.

| **Dataset** | **Number Geneset** | **Thread = 1** | **Thread = 2** | **Thread = 4** | **Thread = 6** | **Thread = 8** | **Thread = 10** |
| --- | --- | --- | --- | --- | --- | --- | --- |
| small | 239 | 6.34 | 4.92 | 4.88 | 5.92 | 7.40 | 9.21 |
| medium | 1000 | 125.41 | 72.96 | 42.31 | 33.57 | 30.54 | 32.01 |
| large | 2287 | 1945.20 | 849.50 | 491.70 | 440.20 | 500.41 | 602.44 |

**Scenarios where “Raw Gene-sets” may be preferred**

While the *“Unique Gene-sets”* method is recommended in most cases for its ability to reduce redundancy and enhance interpretability, there are scenarios where the *“Raw Gene-sets”* method may offer analytical advantages:

- **Comparative analysis across conditions or tools**

In multi-condition or multi-omics studies, users may wish to retain duplicated gene-set entries to inspect how the same pathway appears across multiple comparisons or biological layers. For example, gene-set enrichment might be performed separately on: **Differentially expressed genes (DEGs)** from transcriptomic data (e.g., RNA-seq) and **Peak-associated genes** derived from chromatin accessibility data (e.g., ATAC-seq).

This is especially relevant in matched multi-omic datasets where both transcriptome and chromatin accessibility are profiled in the same single cells (e.g., 10x Genomics Multiome data). Even if both layers highlight the same gene-set (e.g., GO:0007612), the underlying gene contributors may differ due to regulatory or post-transcriptional differences. Treating these as separate entries, as done in the *Raw Gene-sets* approach, allows users to explore such modality-specific pathway activity without premature merging.

- **Tracking variability in gene-set membership**

The same gene set ID may be enriched in multiple experimental contrasts but involve distinct subsets of genes. Preserving each occurrence allows inspection of context-specific drivers and highlights biological nuance within broad functional terms. This can reveal distinct patterns of gene usage within a pathway across stimuli, time points, or conditions.

- **Reproducibility and compatibility**

Analyses originally performed using GeneSetCluster 1.0 relied on the *Raw Gene-sets* methodology. Retaining this mode in version 2.0 ensures that users can replicate prior results or extend earlier projects without altering the structure of the input data.

**Fig. S1. General workflow of GeneSetCluster 2.0.** This diagram shows the main steps of GeneSetCluster 2.0 workflow: harmonization, combining, clustering, annotation, and annotation+.
